# Western diet induces severe nonalcoholic steatohepatitis, ductular reaction, and hepatic fibrosis in liver CGI-58 knockout mice

**DOI:** 10.1038/s41598-020-61473-6

**Published:** 2020-03-13

**Authors:** Pan Yang, Youlin Wang, Weiqing Tang, Weiwei Sun, Yinyan Ma, Shu Lin, Jia Jing, Long Jiang, Hang Shi, Zhiyuan Song, Liqing Yu

**Affiliations:** 1 Department of Cardiology, Southwest Hospital, Army Medical University (The Third Military Medical University), Chongqing, 400038 China; 2 Center for Molecular and Translational Medicine, Institute for Biomedical Sciences, Georgia State University, Atlanta, GA 30303 USA; 3 Department of Animal and Avian Sciences, University of Maryland, College Park, MD 20742 USA; 4 Department of Biology, Georgia State University, Atlanta, GA 30303 USA; 5 Division of Endocrinology, Diabetes, and Nutrition, Department of Medicine, University of Maryland School of Medicine, Baltimore, MD 21201 USA

**Keywords:** Gastrointestinal models, Non-alcoholic fatty liver disease

## Abstract

Humans and rodents with Comparative Gene Identification-58 (CGI-58) mutations manifest nonalcoholic fatty liver disease (NAFLD). Here we show that liver CGI-58 knockout (LivKO) mice fed a Western diet rapidly develop advanced NAFLD, including nonalcoholic steatohepatitis (NASH) and hepatic fibrosis. After 14 weeks of diet challenge, starting at 6 weeks of age, LivKO mice showed increased inflammatory cell infiltration and proinflammatory gene expression in the liver, which was associated with elevated plasma levels of aminotransferases. Hepatic ductular reactions, pericellular fibrosis, and bridging fibrosis were observed only in the LivKO mice. Consistently, the KO mice had a significant increase in hepatic mRNAs for fibrogenic genes. In addition, LivKO mice displayed massive accumulation of lipid droplets (LDs) in hepatocytes. LDs were also observed in the cholangiocytes of the LivKO mice, but not the floxed controls. Four of the five LD coat proteins, including perilipins 2, 3, 4, and 5, were increased in the CGI-58 KO liver. CRISPR/Cas9-mediated knockout of CGI-58 in Huh7 human hepatoma cells induced LD deposition and perilipin expression, suggesting a cell autonomous effect. Our findings establish the Western diet-fed LivKO mice as an animal model of NASH and hepatic fibrosis. These animals may facilitate preclinical screening of therapeutic agents that counter against NAFLD progression.

## Introduction

Nonalcoholic fatty liver disease (NAFLD) is the most common liver disease in the United States and worldwide^1^. A hallmark of NAFLD is hepatic steatosis that is characterized by excessive deposition of triglycerides (TGs)-rich lipid droplets (LDs) in liver as a result of increased synthesis and/or reduced utilization^2^. Simple hepatic steatosis may be relatively benign with little clinical symptoms. However, some individuals with hepatic steatosis have hepatic proinflammatory responses (*i.e*., nonalcoholic steatohepatitis or NASH) and fibrogenesis that can progress to cirrhosis and cancer, causing substantial morbidity and mortality^1,3^. The underlying mechanisms for NAFLD progression remain elusive. Two or multiple “hits” may trigger NAFLD progression by causing hepatocellular injuries, such as elevated production of pro-inflammatory cytokines and reactive oxygen species, fat-induced lipotoxicity, mitochondrial dysfunction, and dysbiosis of gut microbiome^4,5^. Although the etiology of NAFLD is multifactorial, pathological changes in liver are quite similar^6^.

Comparative Gene Identification 58 (CGI-58) is also known as α/β hydrolase domain-containing 5 (Abhd5). It is ubiquitously expressed^7^. Mutations in human CGI-58 cause Chanarin-Dorfman syndrome (CDS), an autosomal recessive neutral lipid storage disease that is characterized by ichthyosis and accumulation of TG-rich cytosolic LDs in all cell types examined^8^. Although no TG hydrolase activity, CGI-58 can function as the coactivator of Adipose Triglyceride Lipase (ATGL)^7^, the rate-limiting enzyme of intracellular LD lipolysis^9^. During lipolysis, ATGL cleaves the first fatty acyl chain from a TG molecule, producing a diacylglycerol (DG) and a free fatty acid. The two acyl chains in a DG molecule are then sequentially removed by Hormone Sensitive Lipase (HSL) and Monoacylglycerol Lipase (MAGL). Mutations in human ATGL also cause an autosomal recessive neutral lipid storage disease^10^.

We have previously shown that mice lacking CGI-58 in liver (LivKO mice) fed a regular chow diet develop NASH and hepatic fibrosis^11^. However, the time needed for manifestation of advanced NAFLD in the chow-fed CGI-58 LivKO mice is quite long^11^, which prevents efficient use of these mice as an animal model of NASH and hepatic fibrosis. Humans do not consume chow that is extremely high in fiber and low in fat and cholesterol. Nutrient compositions are known to have an enomous impact in disease pathogenesis. Western societies consume a diet that typicall has ~40% energy from fat and ~0.2% (w/w) cholesterol. A Western-type diet has been developed to mimic dietary compositions in humans for animal research. Several Western diet-based diets have been used to achieve some features of NAFLD in wildtype rodents, such as a Western-type diet alone, or a Western-type diet with added fructose, free cholesterol, or CCL_4_^12–16^. In this study, we fed LivKO mice with a Western diet and determined whether a nutrient-rich human diet facilitates NAFLD progression in these animals. We showed that LivKO mice on the Western diet rapidly develop the full-spectrum of liver pathologies seen in human NAFLD, including steatosis, NASH, ductular reaction, and fibrosis. Thus, challenging CGI-58 LivKO mice with a Western-type diet may facilitate mechanistic and therapeutic studies of NAFLD progression.

## Results

### Weight gain and hepatomegaly in LivKO mice fed a Western diet

**Figure 1:**
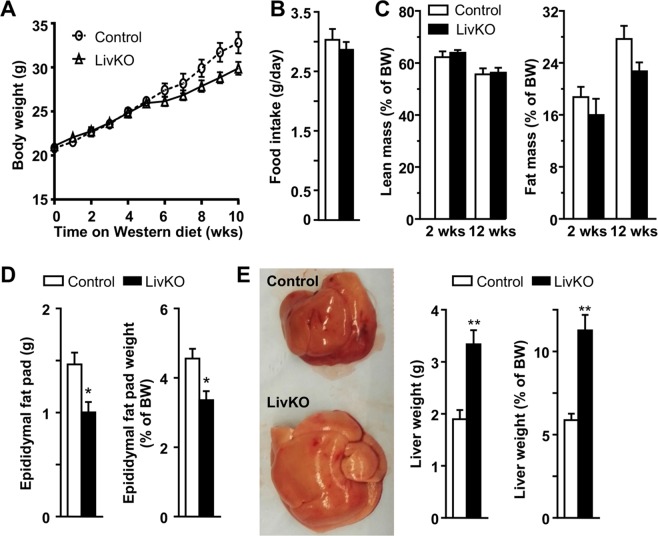
Liver-specific deletion of CGI-58 causes hepatomegaly in male mice. (**A**) Body weight changes of the mice fed the Western diet starting at 6 weeks of age (n = 6). (**B**) Food intake of the mice on Week 13 of Western diet feeding (n = 6). (**C**) Body composition of the mice fed the Western diet for 2 or 12 weeks (n = 6–8). (**D**) Fat weight and fat-to-body weight ratios of the mice at necropsy (n = 6). (**E**) Gross appearance of liver, liver weight, and liver-to-body weight ratios in the mice at necropsy. *P < 0.05; **P < 0.01.

Both male and female LivKO and control mice fed a Western diet gained weight steadily and similarly during the 10 weeks of diet challenge, except that the male KO mice versus controls began to gain less weight after 5 weeks on the diet (Figs. 1A and S1)). It should be emphasized that the KO males did not lose any weight beyond this point, and they instead continued to gain weight steadily (Fig. 1A). Relative to their respective gender controls, both male and female LivKO mice consumed the same amount food (Figs. 1B and S1B), and had the identical lean body mass (Figs. 1C and S1C). The fat body mass was also similar between the two genotypes in both genders after 2 weeks of diet feeding, though the KO mice relative to the floxed controls gained less fat mass after 12 weeks of dietary challenge (Figs. 1C and S1C). Consistently, at the time of necropsy (*i.e*., after 14 weeks on the Western diet, and at the age of 20 weeks), Male and female LivKO mice accumulated less epididymal fat compared to the respective controls (Figs. 1D and S1D). Livers from male and female LivKO mice appeared pale grossly and were significantly enlarged (Figs. 1E and S1E). The liver weight of male and female LivKO mice was significantly increased by 69.1% and 83.5%, respectively, (Figs. 1E and S1E). On average, the liver weight in LivKO mice reached ~11% of body weight, a significant increase from ~6% in control mice (Figs. 1E and S1E).

### LivKO mice fed a Western diet develop severe hepatic steatosis

**Figure 2:**
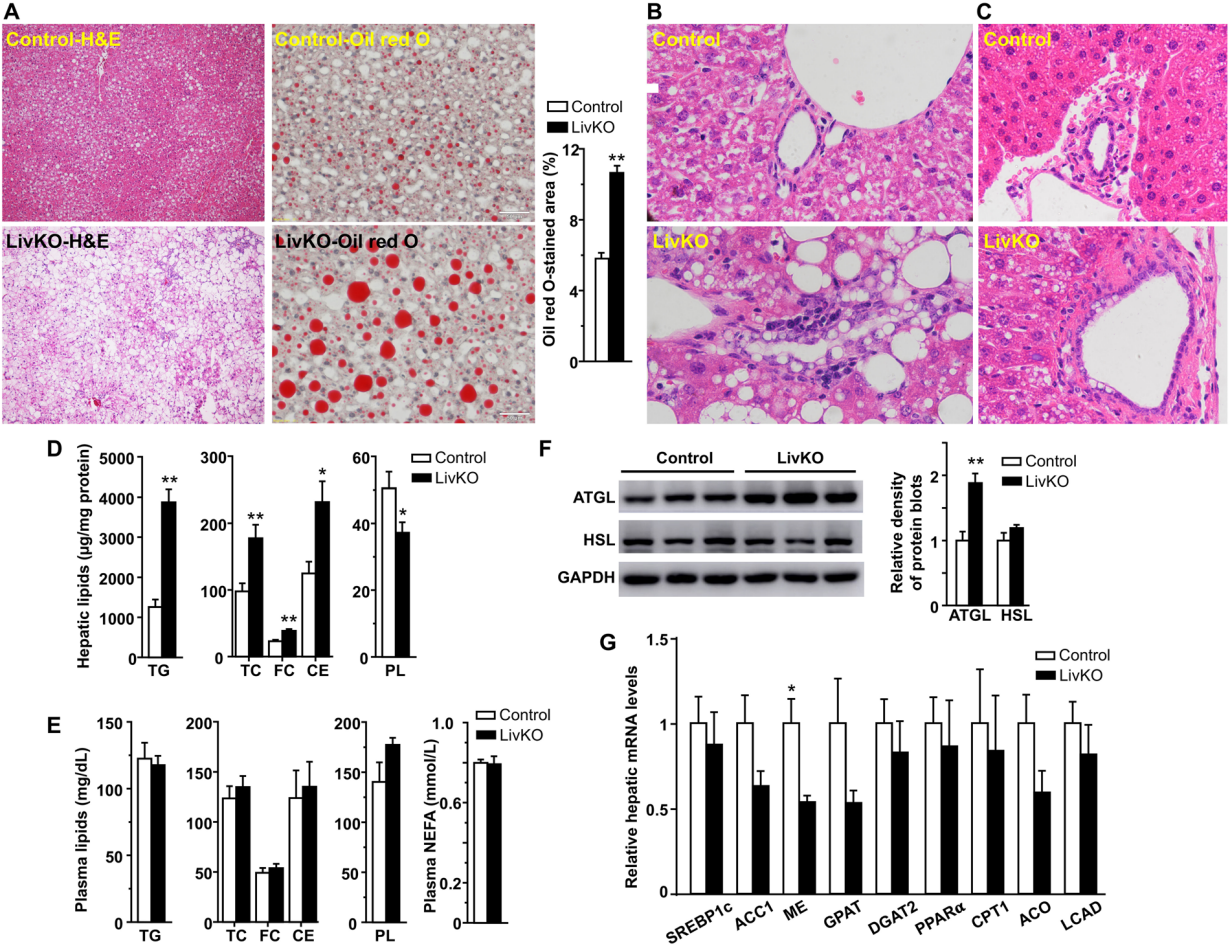
Liver-specific deletion of CGI-58 exacerbates hepatic steatosis in male mice. (**A**) Representative images of H&E and Oil red O staining of the liver sections from the mice at necropsy. The Oil red O-stained area was quantified by ImageJ (n = 12 images per group). Three 20x images from each mouse liver section were taken under an Olympus miscroscope (n = 3 mice per group). (**B**,**C**) H&E staining of liver sections from the mice at necropsy. (**D**) Hepatic contents of triglycerides (TG), cholesterol, and phospholipids (PL) in the mice at necropsy (n = 6). The mass of cholesterol ester (CE) was calculated by multiplying the mass difference between total cholesterol (TC) and free cholesterol (FC) by 1.67. (**E**) Plasma concentrations of lipids in the male mice at necropsy (n = 5–6). (**F**) Immunoblots of hepatic proteins from the mice (n = 5–6 per group) fed the Western diet for 3 weeks. Immunoblots were quantified by densitometry. (**G**) Relative levels of hepatic mRNAs for genes involved in lipid synthesis and oxidation in the mice at necropsy (n = 4–5). GAPDH was used as an internal invariant control. SREBP-1c, sterol regulatory element binding protein-1c; ACC1, acetyl-CoA carboxylase 1; ME, malic enzyme; GPAT, mitochondrial glycerol-3-phosphate acyltransferase; DGAT2, acyl-CoA diacylglycerol acyltransferase 2; PPAR-α, peroxisome proliferator-activated receptor-alpha; CPT-1α, carnitine palmitoyltransferase-1alpha; ACO, acyl-CoA oxidase; LACD, long-chain acyl-CoA dehydrogenase. All samples were from the mice fed the Western diet, except those under (**C**), which were from the mice fed the chow. *P < 0.05; **P < 0.01.

To examine hepatic pathological changes, H&E staining was performed with the liver specimens from LivKO and control mice fed a Western diet for 14 weeks. As expected, Western diet feeding induced hepatic LD deposition in control mice as evidenced by H&E and Oil red O staining of the liver sections (Figs. 2A and S2A). Liver CGI-58 deletion exacerbated this effect. Both microvasicular steatosis and macrovesicular steatosis were observed in LivKO mice (Figs. 2A and S2A). In LivKO but not control mice, LDs were also deposited in the cholangiocytes lining the bile ductules, regardless of diet (Figs. 2B, C and S2B). Biochemically, there were significant increases in hepatic contents of TGs and cholesterol in LivKO mice (Figs. 2D and S2C). Despite substantial changes in hepatic lipids, plasma concentrations of lipids, including TGs, total cholesterol, free cholesterol, cholesterol esters, and nonesterified fatty acids, remained unchanged in both male and female LivKO mice at necropsy (Figs. 2E and S2D).

Liver CGI-58 deficiency did not cause LD deposition by reducing hepatic expression of ATGL and HSL proteins, two lipases responsible for LD lipolysis (Fig. 2F). It instead raised the level of hepatic ATGL protein, suggesting that ATGL is not fully functional without its coactivator CGI-58^7^. It has been well established that CGI-58 deficiency causes cytosolic LD accumulation by inhibiting LD lipolysis^7^, not by promoting lipogenesis, or by inhibiting fat oxidation. Consistently, hepatic mRNAs for several lipogenic genes, including sterol regulatory element-binding protein-1c (SREBP-1c), acetyl-CoA carboxylase 1 (ACC1), malic enzyme (ME), mitochondrial glycerol-3-phosphate acyltransferase (GPAT), and diacylglycerol acyltransferase 2 (DGAT2), were either unaltered or decreased (Fig. 2G). Hepatic mRNAs for genes related to fatty acid oxidation, including peroxisome proliferator-activated receptor alpha (PPAR-α), carnitine palmitoyltransferase-1alpha (CPT-1α), acyl-CoA oxidase (ACD), and long-chain acyl-CoA dehydrogenase (LCAD), were not significantly reduced (Fig. 2G).

### LivKO mice fed a Western diet accumulate LD coat proteins in liver

**Figure 3:**
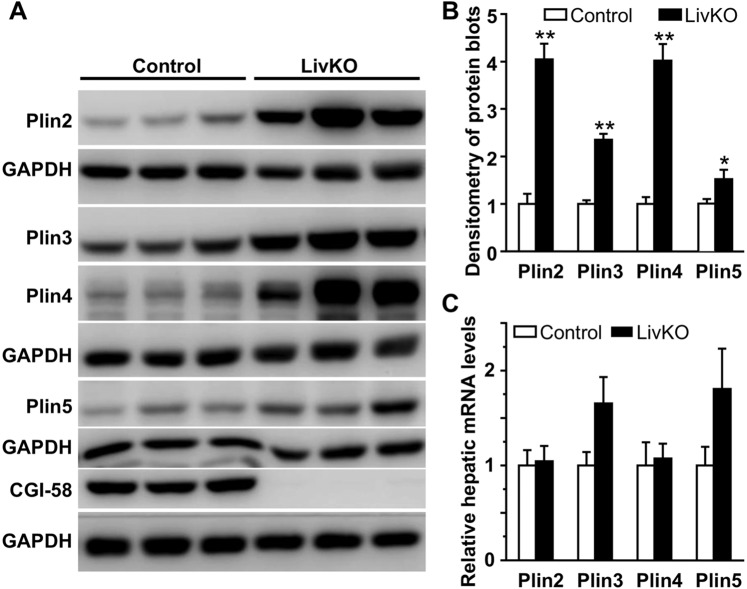
Relative levels of hepatic proteins and mRNAs of PAT family genes in the male mice fed the Western diet for 3 weeks starting at 6 weeks of age. (**A**) Immunoblots of perilipins and control proteins. (**B**) Densitometry of the immunoblots in A (n = 5–6). (**C**) Relative levels of hepatic mRNAs for perilipins in the mice at necropsy (n = 5–6). *P < 0.05; **P < 0.01.

In the Western diet-fed LivKO mice, hepatocytes contained multilocular, and sometimes unilocular, LDs of varying size (Figs. 2A and S2A). Maitenance of cytosolic LD stability likely requires LD structural proteins. Perilipins belong to the PAT (Perilipin, Adipophilin, TIP47) protein family, which has a total of five members, Perilipin 1 (Plin1) to Plin5^17^. They coat the surface of cytosolic LD and serve as the structural compoment of this cellular organelle^18^. Different perilipins predominantly coat LDs of different sizes^19^. Accumulation of LDs is expected to sequester more perilipin proteins on their surface. Increased perilipins appear to play an important role in the pathogenesis of NAFLD in humans^20^. To determine the expression pattern of perilipins in the liver of LivKO mice, hepatic levels of proteins and mRNAs for the five perilipins were determined in the male mice. Plin1 protein and mRNA were undetectable in the liver of both genotypes of mice (not shown). This is not surprising because Plin1 is almost exclusively expressed in adipose tissue^21^. Hepatic Plin2, Plin3, Plin4, and Plin5 proteins were all significantly increased in LivKO mice (Fig. 3A, B). The increase in hepatic Plin2 and Plin4 proteins was not a result of increased transcription because their mRNA levels in liver remained unchanged (Fig. 3C). There was a trend toward an increase in hepatic mRNAs for Plin3 and Plin5 (Fig. 3C).

### CGI-58 deficiency directly induces LD deposition and perilipin expression in Huh7 hepatoma cells

**Figure 4:**
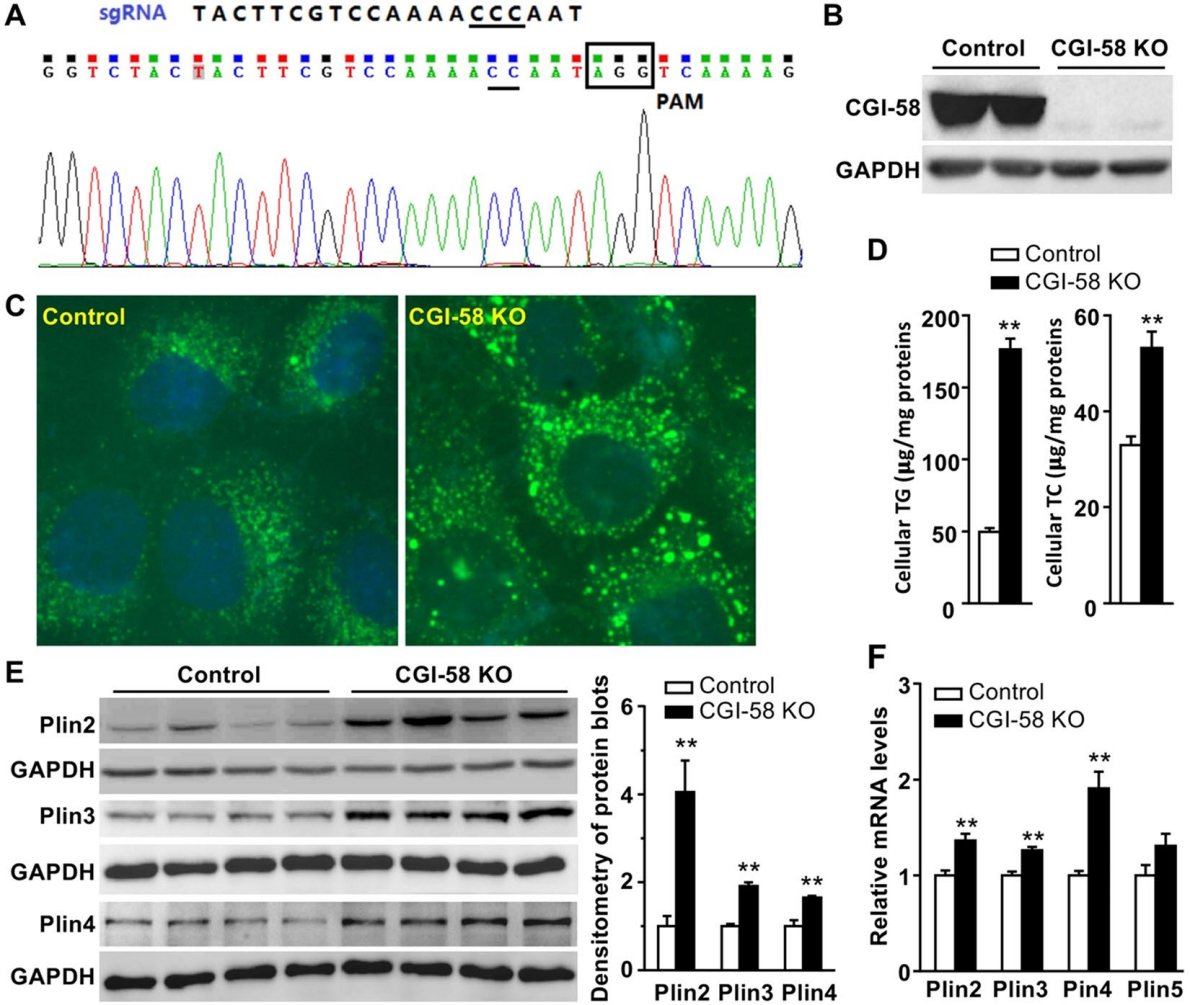
Deletion of CGI-58 in Huh7 human hepatoma cell line increases cellular lipids and perilipin expression. (**A**) The sgRNA sequence targeting Exon 3 of human CGI-58 gene and the sequencing result of the DNA from CGI-58 knockout (KO) Huh7 cells. (**B**) Immunoblots of the KO and control Huh7 cell lysates. (**C**) Fluorescence microscopic images of LDs stained with Bodipy (green) and nuclei stained with DAPI (blue) in the KO and control cells. (**D**) Triglyceride (TG) content in the KO and control cells (n = 5). (**E**) Western blots of perilipins in the KO and control cell lysates. The blots were quantified by densitometry. (**F**) Relative levels of mRNAs for perilipins in the KO and control cells (n = 4). GAPDH was used as an internal invariant control. *P < 0.05; **P < 0.01.

To determine whether alterations in hepatic perilipin expression was a cell autonomous effect of CGI-58 deficiency, CGI-58 was knocked out in Huh7 hepatoma cells using the CRISPR/Cas9 technology (Fig. 4A). Deletion of CGI-58 was confirmed by immunoblotting (Fig. 4B), LD staining (Fig. 4C), and biochemical analysis of total cellular TGs and cholesterol (Fig. 4D). Similar to *in vivo* findings in LivKO mice, Plin2, Plin3 and Plin4 proteins were significantly elevated in the KO cells (Fig. 4E), whereas Plin1 expression was undetectable (not shown). The immunoblot for Plin5 protein was not included due to issues with the antibody. The mRNAs for Plin2, Plin3, Plin4, and Plin5 were moderately elevated in the KO cells (Fig. 4F).

### LivKO mice on a Western diet develop severe NASH

**Figure 5:**
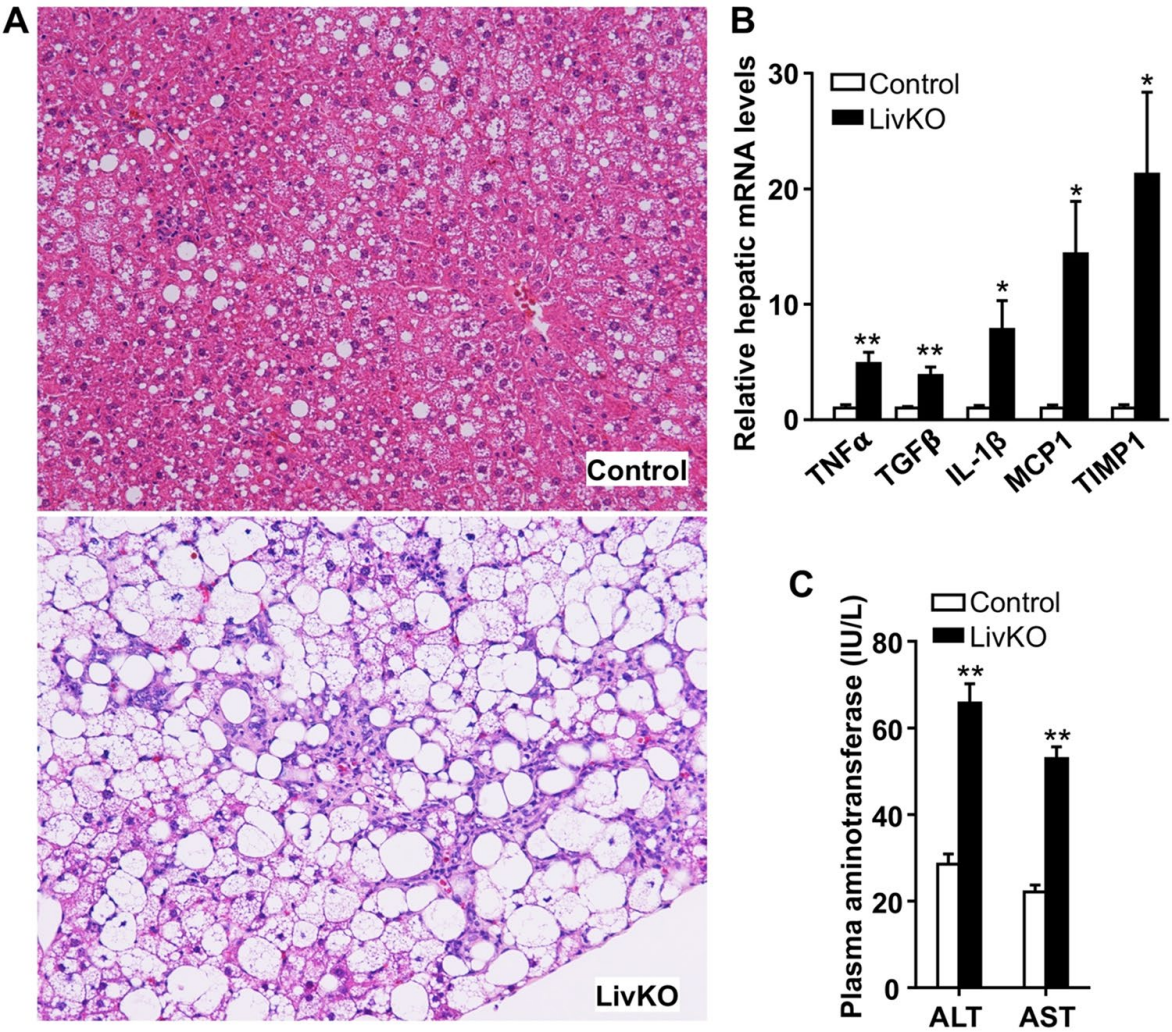
Severe NASH in LivKO mice fed the Western diet for 14 weeks starting at 6 weeks of age. (**A**) H&E staining of liver sections from male mice. (**B**) Levels of hepatic mRNAs for genes related to inflammation in male mice (n = 4–5). GAPDH was used as an internal invariant control. TNF-α, tumor necrosis factor-alpha; TGF-β, transforming growth factor-beta; IL-1β, interleukin-1β; MCP1, monocyte chemoattractant protein 1; TIMP1, tissue inhibitor of metalloproteinase 1. (**C**) Plasma levels of alanine transaminase (ALT) and aspartate transaminase (AST) in male mice (n = 5–6). *P < 0.05; **P < 0.01.

Disease onset is often determined by the interaction between genetic variations and environmental factors, including nutrition. Overnutrition may facilitate, and contribute significantly to, the progression of simple hepatic steatosis to NASH. Only 14 weeks on the Western diet, LivKO mice already displayed substantial lobular and periportal inflammation in liver (Figs. 2A, 5A, S2A and S3A). Their hepatic mRNAs for several proinflammatory genes, including tumor necrosis factor (TNF)-α, transforming growth factor (TGF)-β1, interleukin-1β, and monocyte chemoattractant protein 1 (MCP1), were significantly elevated (Fig. 5B). The hepatic mRNA for tissue inhibitor of metalloproteinase 1 (TIMP1), a cytokine-responsive glycoprotein that inhibits extracellular metalloproteinases (MMPs) to regulate extracellar matrix compositions, was markedly induced in LivKO mice (Fig. 5B). LivKO mice also had a significant increase in plasma concentrations of alanine aminotransferase (ALT) and aspartate aminotransferase, indicative of hepatocyte injuries (Figs. 5C and S3B).

### LivKO mice on a Western diet develop severe hepatic fibrosis

**Figure 6:**
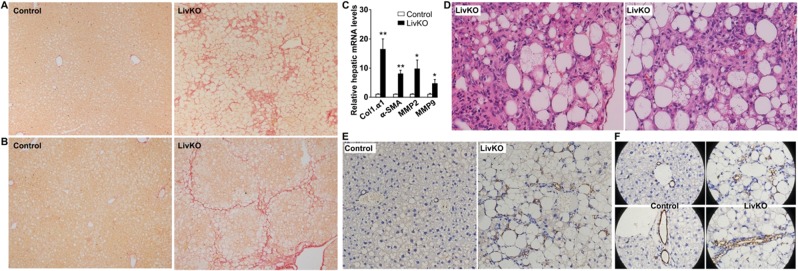
Hepatic fibrosis and ductular reactions in LivKO mice on the Western diet for 14 weeks starting at 6 weeks of age. (**A,B**) Picrosirius Red staining of liver sections from male (**A**) and female (**B**) mice. (**C**) Hepatic levels of mRNAs for genes related to fibrosis (n = 4–5). GAPDH was used as an internal invariant control. Col1α1, collagen type 1 alpha-1; α-SMA, alpha-smooth muscle actin; MMP2, matrix metalloproteinase 2; MMP9, matrix metalloproteinase 9. (**D**) H&E staining of liver sections from male (left) and female (right) mice. (**E**,**F**) Immunohistochemical staining of CK19 in liver sections.

NASH-associated liver injuries activate wound healing mechanisms, which underlies hepatic fibrosis and cirrhosis. Picro-Sirius Red staining of hepatic collagens revealed pericellular and bridging fibrosis, some of which displayed a pseudolobular appearance, in both male and female LivKO mice (Fig. 6A, B). There were marked increases in hepatic mNRAs for markers of fibrosis and tissue matrix remodeling, including collagen type 1 α1 (Col1.α1), α-smooth muscle actin (α-SMA), matrix metalloproteinase 2 (MMP2), and MMP9 (Fig. 6C). We often observed focal scars surrounded by severe steatosis and inflammatory cell infiltration in the liver of LivKO mice, particularly, in the subcapsular region (Fig. 6D).

### LivKO mice on a Western diet show severe hepatic ductular reaction

Hepatic ductular reaction refers to as “a reaction of ductular phenotype, possibly but not necessarily of ductular origin, in acute and chronic liver disease”^22^, including NASH and hepatic fibrosis^23^. To determine if liver CGI-58 deficiency-induced NASH and fibrosis are associated with increased ductular reactions, we performed immunohistochemical studies using the antibody against Cytokeratin-19 (CK19), a marker of cholangiocytes and hepatic ductular reactions. As expected, CK19-positive staining was restricted to the cholangocytes lining bile ducts in the control mice (Fig. 6E). Remarkably, in LivKO mice under the identical dietary condition, CK19-positive ductular reactions were prominent in every microscopic field, periportal regions, in particular (Fig. 6E). The pattern of ductular reaction included an isolated single cell, a cluster of a few cells, and a ring or tubular structure of cells (Fig. 6E,F). Cholangiocytes lining the bile ductules of LivKO mice, but not those of floxed control mice, were filled with vacuoles (Fig. 6E,F), indicative of LD deposition in the biliary epithelial cells of LivKO mice. This was also observed after H&E staining (Figs. 2B,C and S2B).

### Glucose homeostasis and insulin sensitivity in LivKO mice on a western diet

**Figure 7:**
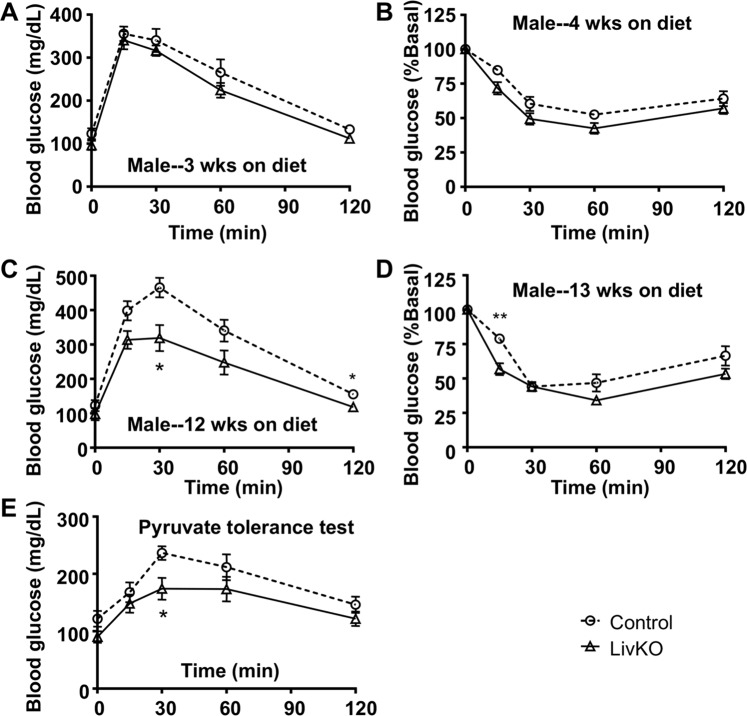
Effects of liver-specific deletion of CGI-58 on systemic glucose tolerance and insulin sensitivity. (**A**,**B**) Glucose tolerance test (GTT) and insulin tolerance test (ITT) in the male mice fed the Western diet for 3 and 4 weeks, respectively, starting at 6 weeks of age (n = 7–8). (**C**,**D**) GTT and ITT in the male mice fed the Western diet for 12 and 13 weeks, respectively (n = 6–8). (**E**) PTT in the male mice fed the Western diet for 13 weeks and fasted for 16 h (n = 7–8). *P < 0.05 and **P < 0.01 (control vs. LivKO at each time point).

Although hepatic steatosis is often associated with insulin resistance^24^, many human subjects with NAFLD have no insulin resistance^2^. To determnine whether liver CGI-58 deficiency-induced NAFLD affects systemic insulin sensitivity and glucose handling, glucose and insulin tolerance tests were performed in the LivKO and control mice fed the Western diet for different durations. When glucose and insulin tolerance tests were performed after 3 and 4 weeks of dietary challenge, respectively, the two genotypes of male mice showed no significant differences in these tests (Fig. 7A, B). When glucose and insulin tolerance was tested after 12 and 13 weeks of diet feeding, respectively, the male LivKO mice versus the male controls did show better glucose tolerance and insulin sensitivity (Fig. 7C, D), but this was the time point when the LivKO males displayed less weight gain when compared to the controls (Fig. 1A). At this time point, the male LivKO mice exhibited reduced gluconeogenesis from pyruvate (Fig. 7E). Female LivKO versus control mice gained weight identically during the entire period of diet challenge, and they showed no differences in glucose tolerance, insulin sensitivity, and pyruvate gluconeogenesis (Fig. S4A–E).

## Discussion

The present study demonstrated that 14 weeks of Western diet feeding induces pronounced hepatic steatosis, NASH, and fibrosis in both male and female mice lacking the lipolytic activator CGI-58 in liver. Our previous and current studies with CGI-58 LivKO mice on different diets collectively suggest that patients with CDS may rapidly develop advanced NAFLD if they consume energy-rich Western-type diets. Avoiding Western-type diets may slow down NAFLD progression in these patients. Interestingly, it was recently shown that CGI-58 is required for Patalin Like Phospholipase Domain Containing 3 (PNPLA3), PNPLA3(I148M) variant, in particular, to promote hepatic steatosis^25,26^. PNPLA3(I148M) is strongly associated with NAFLD and alcoholic fatty liver disease in the population^27–30^. It is the most common genetic risk factor for NAFLD in humans^31,32^. The prevalence of NAFLD induced by CGI-58 monoallelic mutations was estimated to be 1 in 1131 in a normal population^33^. These human genetic studies, together with animal and cell biology data, highlight a critical role of CGI-58 mutations in the etiology of fatty liver disease in the general population. Thus, our animal model is highly relevant to fatty liver disease in humans. It is important to extensively examine how nutritional factors impact NAFLD progression in this animal model. The rapid development of the full spectrum of NAFLD pathologies in CGI-58 LivKO mice on a Western diet will likely facilitate preclinical screening of therapeutic agents against progression of NAFLD, a major health issue worldwide.

In addition to NASH and hepatic fibrosis, another hallmark of the liver pathological changes in LivKO mice on the Western diet is the ductular reaction. Ductular reactions were observed in both acute and chronic liver diseases, including cholestasis, cholangiopathies, NASH, and hepatic fibrosis/cirrhosis^23,34,35^. It is likely a compensatory mechanism to restore liver structures and functions after liver injuries. The origins of ductular reactions may involve lineage conversion, the canals of Hering, and/or stem/progenitor cells in liver^22,23^. In CGI-58 LivKO mice, it is unknown if ductular reactions were caused by the injuries of hepatocytes, cholangiocytes, and/or hepatic stellate cells because the albumin-cre transgenic mouse line used in this study has been shown to delete floxed genes in the three liver cell types^36–42^.

H&E and CK-19 immunohistochemical studies clearly revealed deposition of LDs in both hepatocytes and cholangiocytes in liver CGI-58 KO mice. Liver ATGL KO mice also accumulate LDs in cholangiocytes^43^. One may argue that LD deposition in the cholangiocytes of liver CGI-58 or ATGL KO mice resulted from increased uptake of lipids that were released from neighboring hepatocytes. However, LDs can be observed in the cholangiocytes of the interlobule bile ductules that were not surrounded by LD-containing hepatocytes (Figs. 2B,C and S2B). In addition, LDs were seen in the cholangiocytes of bile ductules in the LivKO mice fed a chow diet, despite modest lipid accumulation in hepatocytes under this experimental condition (Fig. 2C). Moreover, LDs were not observed in the cholangiocytes of control mice fed the Western diet, despite hepatic lipid accumulation (Figs. 2B and S2B). Therefore, the most logical explanation for LD deposition in the cholangiocytes or ductular reaction cells in LivKO mice is that CGI-58 gene was directly excised in these cell types by the albumin promoter-driven cre recombinase. Cholangiocyte and hepatocyte are both derived from hepatoblasts that express albumin. Reactive ductular cells can display cholangiocyte and hepatocyte dual phenotypes^23,44^. Thus, it is not surprising that the albumin promoter-driven cre recombinase can delete floxed genes in both hepatocyte and cholangiocyte. The present findings, together with those in the literature^36–42^, strongly suggest that numerous previous studies using the same albumin-cre mice need to be revisited if the genes of interest are expressed in all liver cells, particularly if liver injuries were induced.

Although CGI-58 is a coactivator of ATGL^7^, liver-specific ATGL knockout mice do not develop NASH and hepatic fibrosis^43^. Mutations in human CGI-58 and ATGL also cause distinct phenotypes. For example, humans with CGI-58 mutations display ichthyosis (scaly dry skin) whereas those with ATGL mutations do not^8,10^. Recently, CGI-58 was shown to function as a coactivator of PNPLA1 in epidermis to maintain the skin barrier function^45,46^. Mutations in human PNPLA1 also cause ichthyosis^47^. Importantly, CGI-58 interacts with PNPLA3, PNPLA3(I148M) variant, in particular^27,48^. PNPLA3(I148M), as the most common genetic risk factor for NAFLD^31,32^, is strongly associated with the full spectrum of pathologies seen in fatty liver disease^27–30^. ATGL is also known as PNPLA2. CGI-58 likely exerts its ATGL-independent functions through interactions with multiple members of the PNPLA protein family. Additionally, CGI-58 interacts with fatty acid binding protein, which may be important in handling of free fatty acids released from LD lipolysis^49^. Intriguingly, CGI-58 was recently shown to possess serine protease activity that cleaves Histone Deacetylase 4 (HDAC4) *in vitro* and in the mouse heart^50^. This study raises several interesting questions. Does CGI-58 cleave a lipase or other lipolysis-regulatory proteins? Is proteolysis a prerequisite for CGI-58 to activate a lipase and promote lipolysis? These versatile functions of CGI-58 provide the basis for the phenotypic differences observed in humans and animals with CGI-58 and ATGL mutations. Detailed analysis of CGI-58 interactome in liver may uncover the mechanisms that drive the progression of simple hepatic steatosis to NASH and hepatic fibrosis.

Cholesterol was accumulated in the liver of CGI-58 LivKO mice fed chow^11^ and Western diets (Figs. 2D and S2C). Hepatic cholesterol content was not reported in the liver ATGL KO mice^43^. Relative to chow, a Western diet is enriched with cholesterol (~0.2%, w/w) besides fat. Hepatic free cholesterol content was 2.3-fold higher in CGI-58 LivKO mice fed the Western diet than the chow diet (Figs. 2D and S2C)^11^. Free cholesterol was shown to cause mitochondrial toxicity in hepatocytes^51,52^, which may be another metabolic cue that triggers rapid development of NASH and fibrosis in LivKO mice fed the Western diet.

Perilipins are structural components of intracellular LDs^17,18^. They are critically implicated in hepatic steatosis^20,53^. Patients with NAFLD display increased levels of hepatic perilipins^53–55^. In the present study, protein levels of hepatic perilipins 2-5 were substantially increased in CGI-58 LivKO mice (Fig. 3A). This increase was likely a cell autonomous effect because similar changes were observed in CGI-58 KO Huh7 cells (Fig. 4). Different perilipins are shown to coat LDs of varying size and compositions^19^. One potential explanation for perilipin accumulation in CGI-58-deficient hepatocytes is that they accumulate passively because all types of LDs can not be utilized without CGI-58. Alternatively, they increase actively to protect cells against lipotoxicity of free lipids. This lipotoxicity scenario seems to be consistent with that antisense oligonucleotide-mediated knockdown of Plin2 increases hepatic expression of fibrogenic genes^56^, though reducing hepatic steatosis^57^. However, liver-specific deletion of Plin2 was reported to reduce hepatic inflammation and fibrosis in mice fed the methionine-choline-deficient diet^58^. A study on clinical specimens revealed a positive correlation between hepatic levels of Plin2 and degrees of hepatic oxidative injuries or hepatocyte ballooning^54^. In addition to Plin2^58,59^, antisense oligonucleotide-mediated knockdown of TIP47 (Plin3), or genetic deletion of Plin5, also reduces hepatic steatosis^60,61^. Global deletion of Plin4 does not seem to significantly reduce hepatic TG^62^. It is unknown if this is due to Plin4’s preference for cholesterol ester-rich LDs^63^. Future studies are required to clarify whether perilipin accumulation is hepatoprotective or hepatotoxic. If hepatotoxic, perilipins may be targeted for prevention of NAFLD development and progression.

While displaying the full spectrum of liver pathologies seen in human NAFLD, CGI-58 LivKO mice relative to the floxed controls fed the Western diet did not show a further increase in insulin resistance, a metabolic disorder that is often associated with hepatic steatosis^24^. Although male LivKO mice relative to floxed controls seemed to handle glucose better after 12–13 weeks of Western diet feeding (Fig. 7), it should be emphasized that LivKO mice fed the Western diet still showed reduced glucose tolerance compared to those fed the chow diet^11^. Additionally, this was a time point when the KO mice versus the controls gained less weight, despite continuing to gain weight (Fig. 1). At this stage, LivKO mice had liver injuries as evidenced by increased plasma AST and ALT levels. They also had severe NASH and hepatic fibrosis. A liver with these severe pathological alterations is expected to have attenuated functions. Consistently, LivKO mice versus controls produced less glucose following pyruvate administration (Fig. 7E). Attenuated gluconeogenesis may reduce insulin resistance, which may cause less increases in blood glucose levels during glucose tolerance tests. This pathophysiological change, however, does not suggest that LivKO mice are more insulin sensitive compared to normal controls. Nonetheless, the dissociation between hepatic steatosis and insulin resistance is not unique to our animal model of NAFLD. For instance, hepatic overexpression of DGAT2, or liver-specific deletion of Histone deacetylase 3 (Hdac3), causes severe TG accumulation in liver, but does not promote insulin resistance in mice^64,65^. In humans, the genetic variant PNPLA3(I148M) confers increased susceptibility to the full spectrum of NAFLD in multiple populations without affecting the index of insulin resistance^27^. African-American descendants have significantly less hepatic steatosis despite a relatively high prevalence of obesity and diabetes whereas Hispanic-American descendants are the opposite^66,67^. The dissociation of hepatic steatosis from insulin resistance among ethnicities seem to suggest that other factors should also be considered^66,67^. Clinical studies of NAFLD only found the association between insulin resistance and hepatic steatosis whereas the relationship between insulin resistance and other pathological changes (NASH, hepatic fibrosis, liver injuries, etc.) has yet to be established.

In conclusion, Western diet feeding induces rapid development of advanced NAFLD in CGI-58 LivKO mice. The liver pathological changes induced by CGI-58 deficiency recapitulates those seen in human NAFLD, including accumulation of perilipin proteins. Rapid establishment of an animal model of NASH and hepatic fibrosis may accelerate mechanistic studies of NAFLD progression and provide a robust *in vivo* system for screening/testing agents that counter against this common liver disease.

## Materials and Methods

### Statement of ethics of animal care and use

All animal procedures were approved by the Institutional Animal Care and Use Committees (IACUC) at Georgia State University. All animal procedures were carried out in accordance with the U.S. National Institute of Health guidelines, including “Principles for Use of Animals” and “Guide for the Care and Use of Laboratory Animals”.

### Mice and diets

CGI-58 LivKO mice were generated as we described previously^11^. Homozygous CGI-58-floxed mice with albumin promoter-driven cre transgene (LivKO) and homozygous CGI-58-floxed mice without this cre transgene (Control) were fed *ad libitum* a standard chow diet (LabDiet) after weaning. Starting at 6 weeks of age, the mice were fed *ad libitum* a Western diet (D12079b, Research Diets, Inc.) for 14 weeks, followed by necropsy. All mice were housed in a specific pathogen-free animal facility at 22 °C with a 12 h light/dark cycle with lights on from 6AM to 6PM. Specific ages of mice used for each experiment are indicated in the legends to figures. Body weight was recorded weekly after initiation of dietary challenge. Body composition was analyzed by EchoMRI-100H Body Composition Analyzer (EchoMRI, LLC.) after Western diet feeding for 2 and 12 weeks.

### Liver histopathology

Liver specimens at necropsy were fixed in the 10% buffered formalin solution and processed for hematoxylin and eosin (H&E) staining. Picro-sirius red staining was done with the paraffin sections by following the instruction of the commercial kit (Catalog #: ab150681, Abcam).

### Determination of plasma, hepatic, and cellular lipids

After a 4 h-fast during the daylight cycle, mice were euthanized. Blood was collected and plasma prepared. Tissues were harvested and snap-frozen in liquid nitrogen for analysis of hepatic lipids as described previously^68,69^. Plasma concentrations of total cholesterol (TC), free cholesterol (FC), and total phospholipids (PLs) were measured by using the TC (Cat. #: 999-02601), FC (Cat. #: 993-02501), and PL (Cat. #: 997-01801) enzymatic assay kits from Wako Diagnostics, Richmond, VA. Plasma concentrations of total TGs were determined by using the enzymatic assay kit from Sigma (Cat. #: F6428 and T2449). Plasma concentrations of nonesterified fatty acids (NEFAs) were determined by using HR Series NEFA-HR Reagents (Cat. #: 999-34691, 995-34791, 991-3489, 993-35191 and 276-76491, Wako Diagnostics, Richmond, VA) following the manufacturer’s instruction. Plasma activities of alanine transaminase (ALT) and aspartate transaminase (AST) were measured by using the commercial kits (Cat. #: A7526, and A7561, Point Scientific, Inc., Canton, MI). For analysis of cellular lipids, the cell pellet from a 10-mm dish with ~80% cell confluence was used. Lipids were extracted twice with two volumes of chloroform:methanol (2:1; v/v) mixture, followed by assays of lipids as described previously^70^.

### Antibodies, immunoblotting, and immunohistochemistry

The monoclonal antibody against CGI-58 was purchased from Abnova (Cat. #: H00051099-M01). This antibody recognizes both human and mouse CGI-58. Plin2 antibody was purchased from Abcam (Cat. #: ab108323). Plin4 antibody (Cat. #: NBP2-13776) and Plin5 antibody (Cat. #: NB110-60509) were obtained from Novusbio. Plin3 antibody (Cat. #: 10694-1-AP) and CK19 antibody (Cat. #: 10712-1-AP) were purchased from Proteintech Group, Inc. (Rosemont, IL, USA). ATGL antibody (Cat. #2138) and HSL antibody (Cat. #: 4107) were purchased from Cell Signaling. GAPDH antibody was obtained from Sigma-Aldrich (Cat. #: G9545) or Proteintech (Cat. # 60004-1-Ig).

For immunoblotting, tissue homogenates or cell lysates were prepared with RIPA Buffer (Cat. #: J62524AD, Alfa Aesar) that contained 10 mmol/L sodium fluoride (Cat. #: S6776, Sigma), 1 mmol/L sodium orthovanadate (Cat. #: 450243, Sigma), and 1% protease inhibitor cocktail (Cat. #: PI78437, Thermo Scientific). A total of 50 μg tissue homogenate proteins per lane or a total of 30 μg of cell lysate proteins per lane was separated by 8% or 10% SDS-PAGE, The proteins in the gel were then transferred to a nitrocellulose membrane. After being blocked with 5% non-fat milk in phosphate-buffered saline (PBS) containing 0.05% Tween 20, the membrane was probed with a primary antibody, followed by a secondary antibody. The membrane was developed with Enhanced Chemiluminescence Detection Reagents (ECL, Thermo Fisher), and then imaged under a Amersham imaging system, or a Bio-Rad imaging system.

Immunohistochemistry was performed with the paraffin-embedded tissue sections. Briefly, epitope retrieval was done for 20 min in an antigen retrieval buffer (10 mM citric acid, 0.05% Tween 20, pH 6.0) that had been heated to 99.2 °C in a water bath. Peroxidase blocking solution (3% H_2_O_2_ in PBS) was then applied to the sections for 20 min at room temperature, followed by incubating with the goat serum blocking solution (2% goat serum and 0.3% Triton X-100) for 60 min. The tissue sections were then incubated in a primary antibody against CK19 (1:2000 dilution) for 30 min at 37 °C and overnight at 4 °C, followed by incubation with a biotinylated secondary antibody and the streptavidin-HRP for 30 min at room temperature. The sections were then developed with diaminobenzidine solution (DAB) for 1–3 min.

### RNA extraction and quantitative real-time PCR

Total RNAs from tissue samples or cell pellets were isolated by using the TRIzol Reagent (Invitrogen). The cDNAs were reversely transcribed from 2 μg of total RNAs using TaqMan Reverse Transcription Reagents (Applied Biosystems). Expression levels of mRNAs were determined by quantitative real-time PCR (qPCR) using Applied Biosystems 7500 qPCR machine. Reactions were done in triplicate for each sample using SYBR Green Real-Time PCR Master Mix (Invitrogen). The relative expression level of each mRNA was calculated by the 2(^−DDCt^) method. GAPDH was used as an internal invariant control. All qPCR primer sequences are available upon request.

### Cell culture and creation of CGI-58 KO Huh7 human hepatoma cell line

Huh7 human hepatoma cells were incubated under humidified air containing 5% CO_2_ at 37 °C and grown to subconfluence in Dulbecco’s modified Eagle’s medium (DMEM) supplemented with 10% fetal bovine serum (FBS), 100 mg/ml penicillin and 100 mg/ml streptomycin. Typically, cells were seeded and grown for 24 h to 70–80% confluence before an experiment. CGI-58 gene was deleted in Huh7 cells using the CRISPR/Cas9 technology described by Dr. Zhang’s group^71^. Briefly, the single guide RNA (sgRNA) targeting at Exon 3 of CGI-58 gene was designed using http://crispr.mit.edu/ website. After synthesized by IDT (Integrated DNA Technologies, Inc.), the sgRNA was inserted into the Bbsl site of the plasmid pSpCas9(BB)-2A-GFP (Plasmid #: 48138, Addgene). The sgRNA-containing plasmid was tranfected into Huh7 cells using SuperFect Transfection Reagent (QIAGEN). The GFP-positive cells were sorted by Flow Cytometry and seeded in 96-well plates with 200 μl of culture medium in each well. When cell colonies were formed in 96-well plates, they were digested by 0.25% Trypsin (HyClone™) and passaged to 48-, 24-, 12-, and 6-well plates successively. CGI-58 KO Huh7 cells were identified by sequencing and Western blotting. Since CGI-58-deficient cells accumulate LDs, the KO cells were also visualized under a fluorescent microscope after LD staining. For this purpose, the cells were seeded on 18×18 mm cover slips and grown for 24 h to ~50% confluence. After LD staining with BODIPY 500/510 (Cat. #: D3823, Invitrogen), the cells were fixed in 4% paraformaldehyde for 10 min. The coverslips were mounted with the ProLong Gold Antifade Mountant with DAPI (Cat. #: P36931, Invitrogen).

### Glucose, insulin, and pyruvate tolerance tests

For glucose tolerance tests, mice were fasted overnight (~16 h). Each mouse was then weighed. After measuring the baseline level of blood glucose via a tail nick using OneTouch Ultra Test Strips and a Glucometer (Lifescan Canada Ltd.), a 20% glucose solution was injected intraperitoneally at 1.5 mg/g BW. Blood glucose levels were measured at 15, 30, 60, and 120 min after glucose injection. For insulin tolerance tests, mice were fasted for 6 h during the daylight cycle. After measuring the baseline level of blood glucose, mice were injected intraperitoneally with the recombinant human insulin at 1.0 mU/g BW. Their blood glucose levels were measured at 15, 30, 60, and 120 min post insulin administration. For pyruvate tolerance tests^72,73^, mice were fasted overnight (~16 h). After measuring the baseline level of blood glucose, a 20% pyruvate sodium solution were injected intraperitoneally at 1.5 mg/g BW. Blood glucose levels were then measured at 15, 30, 60, and 120 min post pyruvate injection.

### Statistical analysis

Data are expressed as the Mean ± SEM. All data were tested for statistical significance by using the two-tailed Student’s *t*-test. A *p*-value less than 0.05 was considered to be statistically significant. Statistical analysis was performed using SPSS 21.0 Software.

## Supplementary information

Supplementary information.

## Data Availability

The data generated and/or analysed during the current study are available from the corresponding authors on reasonable request.
