# Supplementary material for: Western diet induces severe nonalcoholic steatohepatitis, ductular reaction, and hepatic fibrosis in liver CGI-58 knockout mice
Source: Sci Rep. 2020 Mar 13;10:4701. doi: 10.1038/s41598-020-61473-6 (PMC7070035; doi:10.1038/s41598-020-61473-6)
Supplement: Supplementary file 1 — Supplementary information. [file 41598_2020_61473_MOESM1_ESM.pdf]

SUPPLEMENTAL DATA

Western diet induces severe nonalcoholic steatohepatitis, ductular reaction, and hepatic fibrosis in liver CGI-58 knockout mice

Pan Yang[1,2,3], Youlin Wang[2,3], Weiqing Tang[3], Weiwei Sun[1], Yinyan Ma[3], Shu Lin[1], Jia Jing[4], Long Jiang[5], Hang Shi[4], Zhiyuan Song[1*], and Liqing Yu[2,3,5*]

[1]Department of Cardiology, Southwest Hospital, Army Medical University (The Third Military Medical University), Chongqing, 400038, China; [2]Center for Molecular and Translational Medicine, Institute for Biomedical Sciences, Georgia State University, Atlanta, GA 30303, USA; [3]Department of Animal and Avian Sciences, University of Maryland, College Park, MD 20742, USA; [4]Department of Biology, Georgia State University, Atlanta, GA 30303, USA; and [5]Division of Endocrinology, Diabetes, and Nutrition, Department of Medicine, University of Maryland School of Medicine, Baltimore, MD 21201, USA.

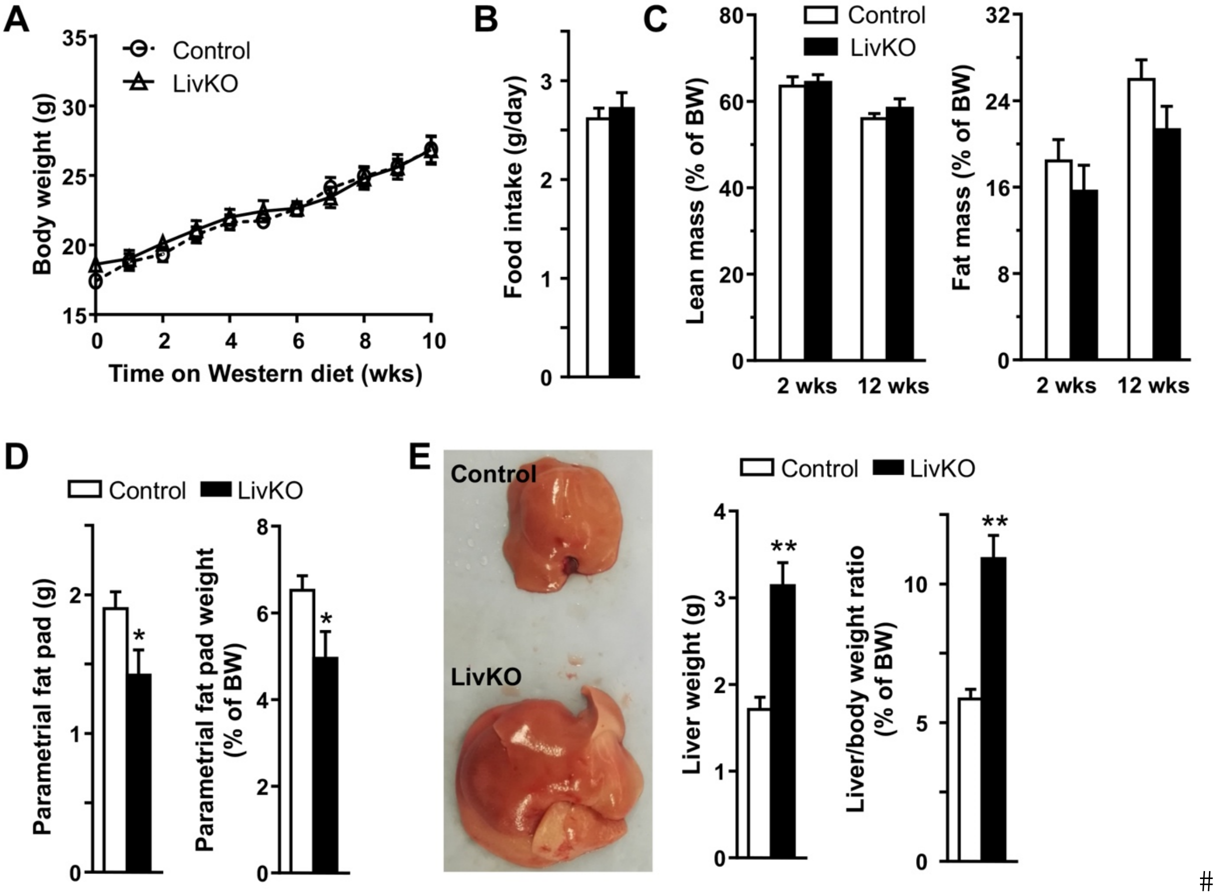

#

Figure S1. Liver-specific deletion of CGI-58 causes hepatomegaly in female mice. A: Body weight changes of the mice fed the Western diet starting at 6 weeks of age (n = 6). B: Food intake of the mice on Week 13 of Western diet feeding (n = 6). C: Body composition of the mice fed the Western diet for 2 or 12 weeks (n = 6-8). D: Fat weight and fat-to-body weight ratios of the mice at necropsy (n = 6). E: Gross appearance of liver, liver weight, and liver-to-body weight ratios in the mice at necropsy. *P < 0.05; **P < 0.01.

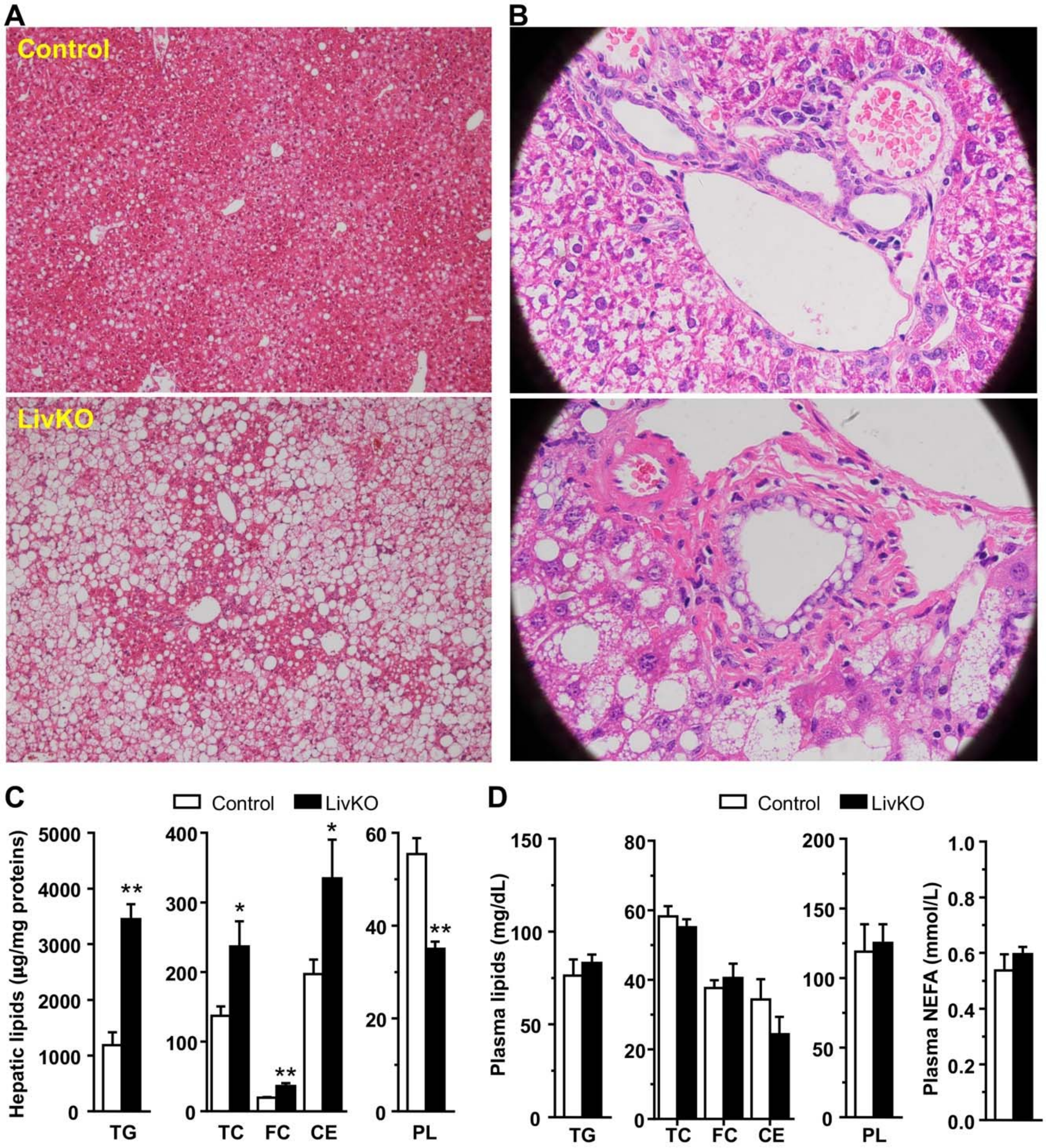

Figure S2. Liver-specific deletion of CGI-58 exacerbates hepatic steatosis in female mice fed the Western diet. A,B: H&E staining of liver sections from the mice at necropsy. C: Hepatic contents of triglycerides (TG), cholesterol, and phospholipids (PL) in the mice at necropsy (n = 6). The mass of cholesterol ester (CE) was calculated by multiplying the mass difference between total cholesterol (TC) and free cholesterol (FC) by 1.67. D: Plasma concentrations of lipids in the mice at necropsy (n = 6). *P < 0.05; **P < 0.01.

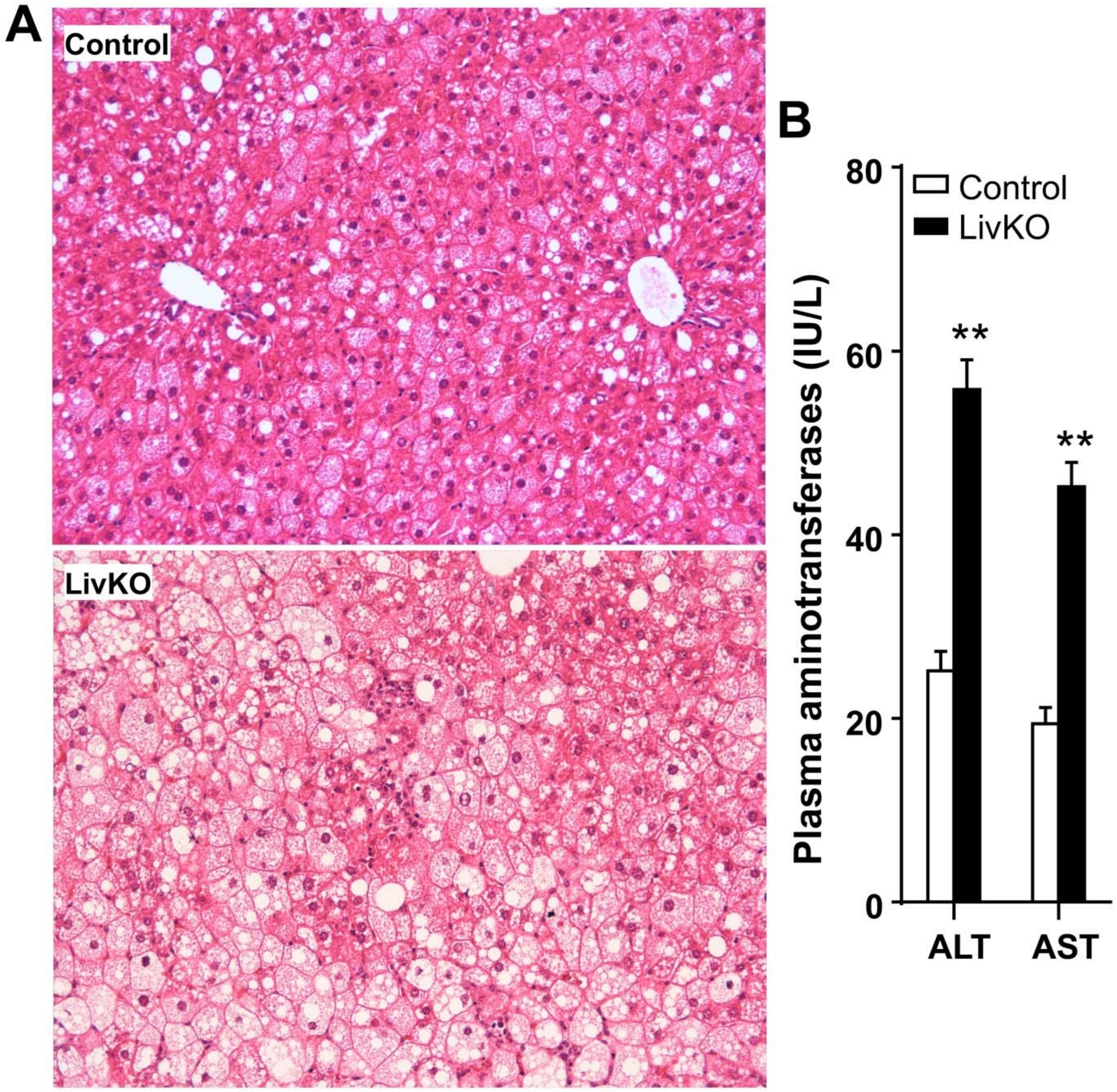

Figure S3. NASH in LivKO female mice on the Western diet for 14 weeks starting at 6 weeks. A: H&E staining of liver sections. B: Plasma levels of alanine transaminase (ALT) and aspartate transaminase (AST) (n = 6). **P* < 0.05; ***P* < 0.01.

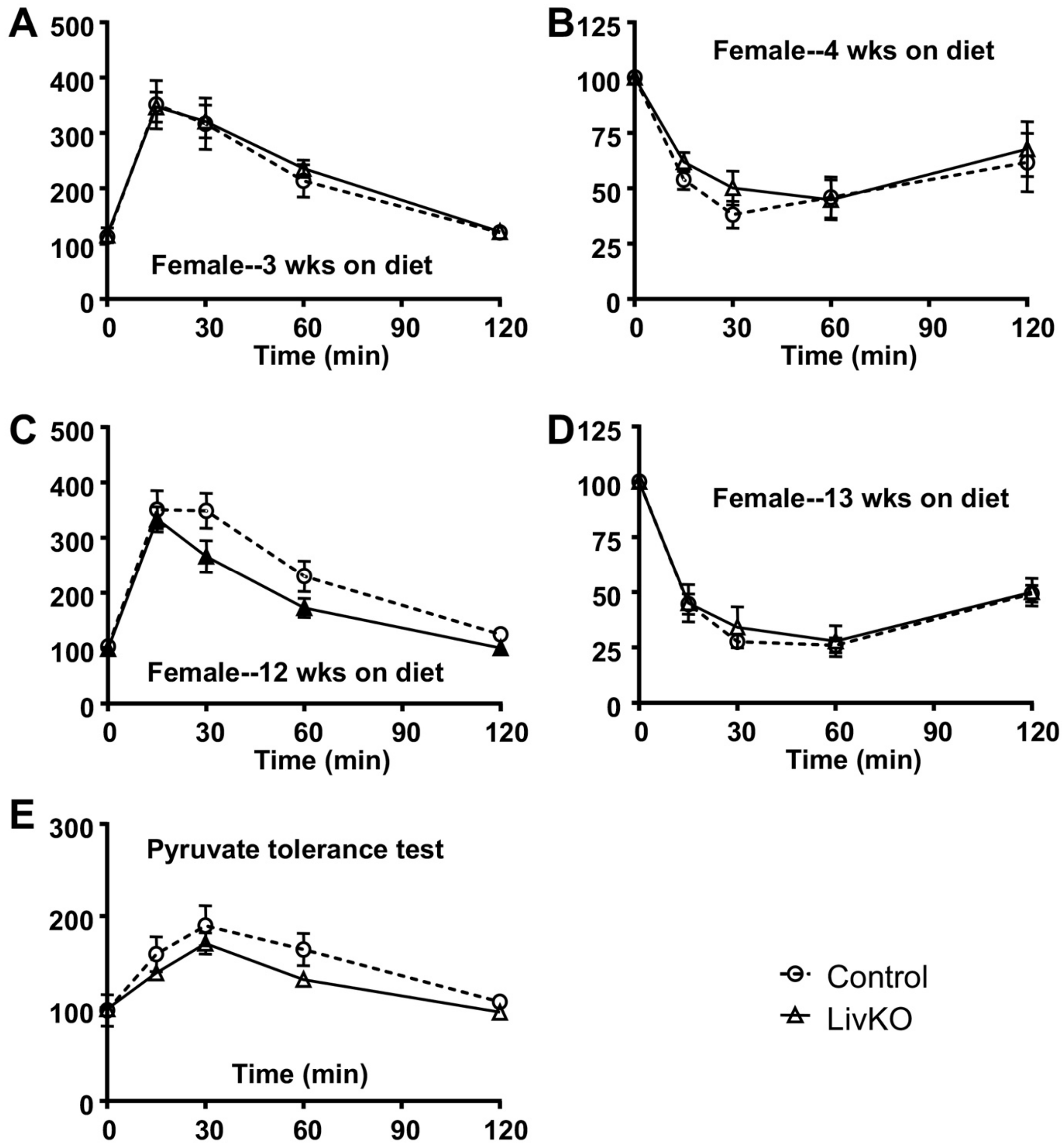

Figure S4. Liver-specific deletion of CGI-58 does not affect systemic glucose tolerance and insulin sensitivity in female mice. A,B: GTT and ITT in the mice fed the Western diet for 3 and 4 weeks, respectively, starting at 6 weeks of age (n = 6-8). C,D: GTT and ITT in the mice on the Western diet for 12 and 13 weeks, respectively (n=6-8). E: PTT in the mice fed the Western diet for 13 weeks and fasted for 16 h (n = 6-8). *P < 0.05 and **P < 0.01 (control vs. LivKO at each time point).
